# Air-Writing Character Recognition with Ultrasonic Transceivers

**DOI:** 10.3390/s21206700

**Published:** 2021-10-09

**Authors:** Borja Saez-Mingorance, Javier Mendez-Gomez, Gianfranco Mauro, Encarnacion Castillo-Morales, Manuel Pegalajar-Cuellar, Diego P. Morales-Santos

**Affiliations:** 1 Infineon Technologies AG, Am Campeon 1-15, 85579 Neubiberg, Germany; borja.saezmingorance@infineon.com (B.S.-M.); Javier.MendezGomez@infineon.com (J.M.-G.); Gianfranco.mauro@infineon.com (G.M.); 2 Department of Electronic and Computer Technology, University of Granada, Avenida de Fuente Nueva s/n, 18071 Granada, Spain; encas@ugr.es; 3 Department of Computer Science and Artificial Intelligence, University of Granada, Calle Periodista Daniel Saucedo Aranda s/n, 18071 Granada, Spain; manupc@ugr.es

**Keywords:** ultrasound, air-writing, gesture recognition, deep learning

## Abstract

The interfaces between users and systems are evolving into a more natural communication, including user gestures as part of the interaction, where air-writing is an emerging application for this purpose. The aim of this work is to propose a new air-writing system based on only one array of ultrasonic transceivers. This track will be obtained based on the pairwise distance of the hand marker with each transceiver. After acquiring the track, different deep learning algorithms, such as long short-term memory (LSTM), convolutional neural networks (CNN), convolutional autoencoder (ConvAutoencoder), and convolutional LSTM have been evaluated for character recognition. It has been shown how these algorithms provide high accuracy, where the best result is extracted from the ConvLSTM, with 99.51% accuracy and 71.01 milliseconds of latency. Real data were used in this work to evaluate the proposed system in a real scenario to demonstrate its high performance regarding data acquisition and classification.

## 1. Introduction

Air-writing is a particular case of gesture recognition. The user draws in the air the character or word to recognize, and the system performs the tracking of the movement and matches the drawn with the actual character or word [1,2]. Air-writing systems present several challenges, such as the lack of a physical writing plane (gestures performed in an imaginary plane) and the detection of starting and ending points of the drawn character. In addition, these systems present a lack of visual feedback when a sequence of tracks is performed, thus increasing the recognition task complexity.

Air-writing systems are described in the literature using different technologies. There are systems based on video [3], infrared (IR) sensors [4,5], radar [6,7,8], Wi-Fi signal [9], RFID [10], or a combination of those technologies (i.e., IR sensors and video [11,12]). There are also works based on ultrasound technology such as Chen H. et al. [13], who proposed a system where the recognition is based in a fixed receiver array performing the localization of a transmitter array attached to the user’s hand. Similarly, Chen J. et al. [14] describe the use of ultrasonic signal and radio signal together to develop a transmitter 3D-pen, and the algorithm to positioning it based on a set of receiver nodes.

The aim of this work is to propose a new air-writing system based on only one array of ultrasonic transceivers, which will perform both emitter and receiver roles. This array will remove the necessity of any active part used by the person performing the character track. This track will be calculated based on the pairwise distance of the hand marker with each transceiver.

**Figure 1:**
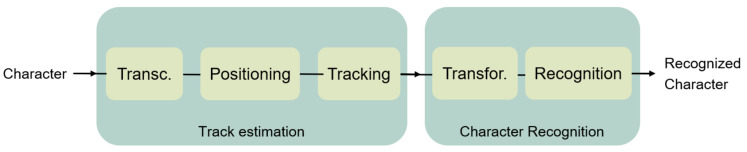
General block diagram for air-writing systems.

The development of the air-writing system can be depicted as the study of two individual tasks, as shown in Figure 1. The first task consists of the estimation of the character drawn by the user. After the track is estimated, in the second task, the recognition of the character will be performed. To do so, the recognition algorithm may execute necessary transformations to the track.

The first task starts with signal acquisition and analysis. While this is highly technology-dependent, the following steps may use common approaches. The hand marker location can be based on a method such as Time of Flight (ToF) [15], Time Difference of Arrival (TDoA) [16], Direction of Arrival (DoA) [17], Angle of Arrival (AoA) [18], etc. After the parameter calculation, the actual position has to be computed. The preferred method in the literature to determine the position is Trilateration (or its generalization for multiple nodes, Multilateration) [19]. The system described in this paper is based on the ToF method to acquire the hand marker position. The 3D position is determined by using multidimensional scaling (MDS) and optimization algorithms, as Limited-Memory Broyden Fletcher Goldfarb Shanno (LM-BFGS) algorithm. Those algorithms have been proved to deal with the ultrasonic noise figure on air, needing a filtering step to remove possible outliers afterwards [20].

The second task is to perform the characters recognition. The track estimated in the previous steps may need to be transformed to fit the characteristic of the recognition algorithms. There is a huge variety of algorithms in the literature for this purpose. As examples, Arsalan et al. [6] transform the three-dimensional estimated track and in a two-dimensional track, which will be fed into a Neural Network to perform the recognition; Leem et al. [8] convert the point-based track obtained from the radar signal to an actual image, using image processing techniques to obtain the written character.

In this work, multiple classification algorithms have been tested on ultrasound-based gesture recognition to determine their suitability. To be precise, these Deep Learning (DL) algorithms are Convolutional Neural Networks (CNN), Long Short-Term Memory Neural Networks (LSTM NN), Autoencoders, and variations of these algorithms. These algorithms have been selected according to the high-accuracy results achieved by other authors for gesture recognition tasks [6,8,9].

The use of ultrasound technology mitigates the disadvantages that other technologies present. Image-based systems (cameras or IR sensors) are affected by changes in the ambient light conditions, and they may also lead to privacy issues related to the identification of users. Radio-based systems (such as Wi-Fi or radar), thanks to intrinsic technology characteristics, could overcome these problems. These systems could suffer from signal interference regardless due to the increasing number of applications based on this technology. Finally, the use of active wearable-based systems (such as ultrasound transmitters) can lead to a more complex solution, which would consequently be less intuitive for users.

This work is structured as follows: Section 1 introduces the state of the art. Section 2 and Section 3 explain in detail the algorithms studied in this work for the track acquisition and classification, respectively. Section 4 presents the dataset used in this work and the specific parameters used for each of the classification algorithms. Section 5 summarizes the results obtained. Finally, Section 6 focuses on the conclusions of this work.

## 2. System Description

**Figure 2:**
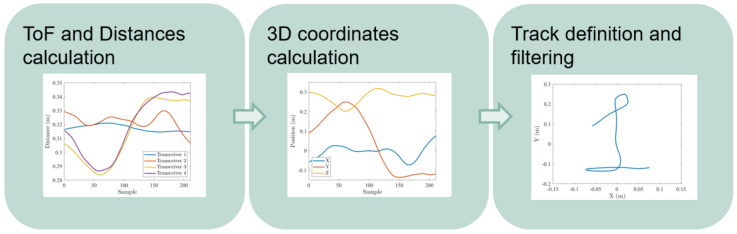
Track generation pipeline.

This section covers the detection of the user movements and the translation into a temporal series of positions. It is divided into three tasks, covering the movement sensing, individual position calculation and the complete track estimation, following the steps shown in Figure 2.

### 2.1. Hardware

**Figure 3:**
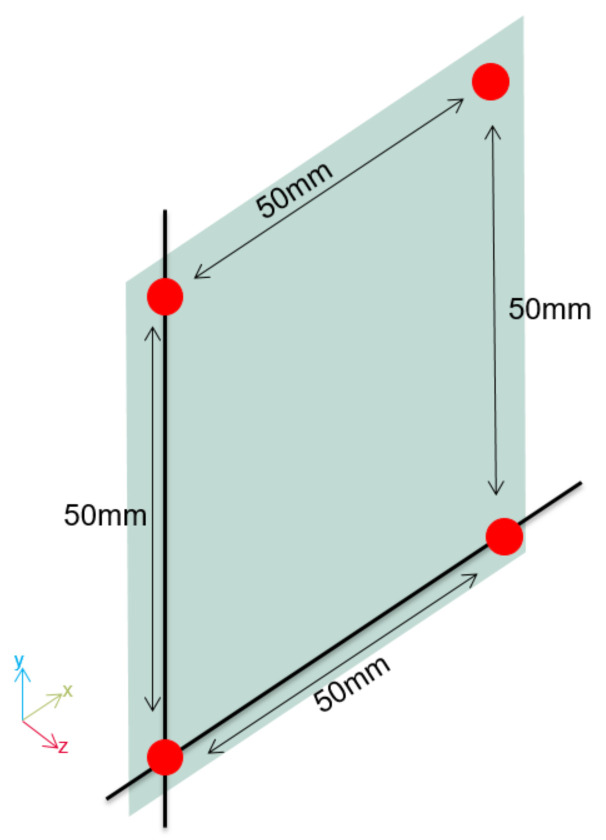
Position of the ultrasonic transceivers.

For user detection, this work uses four dual-backplate MEMS microphone-based ultrasonic transceivers [21] in a square-shaped matrix, as shown in Figure 3. The transceivers need low bias voltage and support the use of both audio microphones (with a 68 dB(A) signal-to-noise (SNR) performance) and an airborne ultrasonic transceiver (with between 80 and 90 dB SNR). The use of the transceiver to emit an ultrasonic pulse produces a shadow zone of about 10 cm due to the free oscillation of the membrane (ringing).

For the transceivers’ actuation and read-out, the Analog Discovery 2 (AD) was used [22]. As each AD has two analog input channels, it is necessary to use two devices to acquire the output signal of the four transceivers. The AD waveform generator allows the creation of arbitrary signals, used for the transceiver actuation. The parameters used for the transceivers’ actuation and data acquisition are listed in Table 1. The parameter selection is based on results extracted from previous works [23].

#### 2.1.1. Signal Model/Target Detection

As stated in Section 1, the system is based on the time elapsed between the signal emission and the echo reception, known as ToF. In the literature, numerous methods can be found based on techniques such as biologically inspired algorithms [24], based on phase difference [25] or frequency difference [26]. Most works use a method based on cross-correlation and threshold power because of the low computational power required and the noise influence removal effect of the cross-correlation, which acts as matched filtering. Using a template of the expected echo and performing the correlation with the acquired signal, this method produces a time–domain signal with a peak value when the actual echo is received [14,27].

For this work, the ToF will be obtained through a cross-correlation algorithm and a dynamic threshold method. The target distance is then obtained from the ToF. This algorithm locates the closest target that generates the first echo. Obstacles located further than the relevant target are not detected as part of the gesture. The process can be divided into four steps [23]:

1. Cross-correlation. The acquired signal is cross-correlated with a template containing the expected echo. This method will give a maximum value in the sample where the template and the acquired signal match.
2. Dynamic Threshold. In order to distinguish whether there is an echo or not, the value of the cross-correlated signal needs to be greater than a threshold level. The dynamic threshold used in this step decrease the value with the time, to match the attenuation of the signal with the distance traveled [28]. This parameter can be increased or decreased to fit certain conditions, i.e., ambient noise. The cross-correlated signal obtained in the previous step is filtered to extract the envelope, and this envelope is then evaluated to check if and where it crosses the threshold level.
3. ToF calculation. All previous calculations are done over the sample number. When the crossing point between the cross-correlation envelope and threshold is calculated, the sample can be converted to time using the ADC sampling frequency parameter.
4. Distance calculation. Once the ToF is calculated, it can be converted to distance using the following equation [29]:
  (1) $$d=\frac{ToF\;c_{s}}{2}$$
  where *d* is the distance between the hand marker and the transceiver, ToF indicates the ToF calculated, and $c_{s}$ is the speed of sound.

#### 2.1.2. Object Positioning

**Figure 4:**
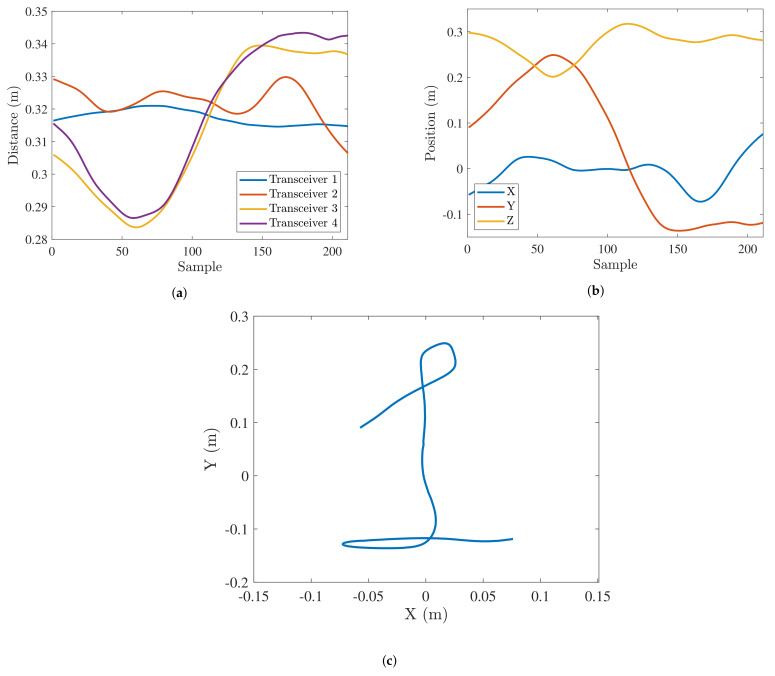
Different steps in the track estimation: Pairwise distances among transceivers and hand marker (**a**), 3D position time series (**b**), 2D projection (**c**).

Once the pairwise distance between the hand marker and each anchor has been obtained as explained in the previous section, those values can be fed to the algorithm to determine the 3D space position, as shown in Figure 4a.

As described in Section 1, in this work a novel algorithm [20] will be used, instead of Multilateration. This method proposes a two-step algorithm to obtain the hand marker location. The first step performs the anchor location, and the second step calculates the hand marker location based on the previously calculated anchors position and the pairwise distances. As the distances among the anchors are known, the first step can be repeated with low frequency to check whether the previously calculated anchor positions are still valid or not. The hand marker position must be calculated with every new sample (each 20 ms as explained in Table 1). The 3D position will be calculated using the LM-BFGS algorithm, minimizing the mean squared error as the objective function.

#### 2.1.3. Track Definition and Filtering

Based on the algorithms previously described, and performing the position estimation periodically, the trajectory of the hand marker is built as a discrete time series of successive points, as shown in Figure 4b. The knowledge of parameters like the maximum speed of the movement and the time between samples makes possible the use of filters. These filters can be used to smooth the trajectory, remove the effect of outliers and restore missing points by interpolation. In this work, the smoothing will be based on the moving average filter algorithm (EQ), which has been proven to reconstruct the original gesture [20].

(2) $$y\left[n\right]=\frac{1}{M}\sum_{j=\frac{-(M-1)}{2}}^{\frac{\left(M-1\right)}{2}}x\left[n+j\right]$$

where X is the input signal, Y is the output, and *M* is the window size—*M* being odd. This filter behaves as a low-pass filter but is focused on time-based response instead of frequency-based. The bigger the window size (greater *M*), the stronger the noise reduction but the greater the delay introduced by the filter as well. Therefore, the value M has to be a trade-off to remove the noise but also to be able to respond to faster movements [30].

#### 2.1.4. Track Transformations

Before using the gathered data for gesture recognition, the data have been adapted to the required format for each of the researched classification algorithms. The first step in the preprocessing pipeline was the projection of the 3D gestures into a 2D plane, as depicted in Figure 4c, to generate 2D images that can be fed into the CNN, ConvLSTM and the convolutional autoencoder. The selected 2D plane was the XY plane as the variance of the gesture with respect to the Z-axis is much smaller than the variance in the other axis. These images were later normalized and converted to grayscale to reduce their dimensions while maintaining relevant features. Due to the nature of the data studied in this research, data augmentation techniques have been applied to the image in addition to 3D coordinate data, as described in Section 4.1.

## 3. Character Recognition Algorithms

Multiple Deep Neural Networks (DNN) are examined for character recognition based on the previously generated trajectory data. The trajectory can be represented as an image or as the 3D numerical coordinates depending on the data type required for each algorithm.

**Figure 5:**
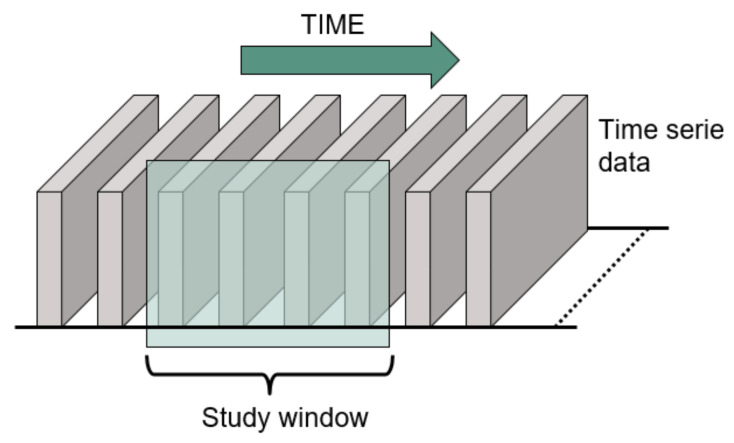
Sliding window used when studying time series with LSTM.

Using these two datasets (images and 3D coordinates), a large range of DNN models can be trained for the classification of the gestures. The most relevant DNN structures in gesture recognition have been selected to classify our data due to previous high-performance results [3,4,6,7,8,9,13,31,32,33]. Different DNN approaches are included in this work to also compare the effect in the classification of the two previously depicted data types. The compared algorithms are:

- Convolutional Neural Network. This DNN model is based on a set of convolutional filters that are applied sequentially to the input data to generate feature maps. The bias and kernel values of these filters are calculated during the training phase of the model. The features extracted with these convolutional filters are later used by fully connected layers for classification or prediction tasks as a traditional Multilayer Perceptron would do. In this work, the CNN will be used to classify the input data as one of the possible studied characters. The input data fed into the CNN are the final 2D-images where the whole characters are represented. Consequently, this DNN is trained to classify each input data point individually without taking into account the length of time of each character or the time distribution of the positions.
  This DNN structure was selected according to the high-accuracy results achieved in the literature for gesture recognition [3,6,8,9]. These works focus on gesture data recorded with multiple sensors such as radar or Wi-Fi. Because of this, it is desired to research if similar results can be achieved when using ultrasound data.
- Convolutional Autoencoder. The convolutional autoencoder can be employed to extract features from data in an unsupervised fashion [34]. This DNN consists of two main parts: an encoder, which maps the images into an embedded representation called code, and a decoder that reconstructs the original image from the code. Therefore, the encoder and decoder can be trained by using the same data as input data and expected output. The Autoencoder can also be combined with convolutional filters for efficient data coding when a more complex feature extraction is required. As for CNN, the encoder can be the input of fully connected layers for the classification of features in different categories. The use of encoders in classification tasks can bring several benefits such as dimensionality reduction and performance improvements in supervision [13,31,32,33]. As with CNN, the inputs fed into the convolutional autoencoder are the final 2D images where the whole characters are represented. Therefore, no time information is considered.
- Long Short-Term Memory (LSTM) DNN. This DNN structure focuses on studying temporal features of the input data by studying its evolution during a selected period of time following a window approach, as shown in Figure 5. The main characteristic of this structure lies in the fact that the output of a hidden layer is transferred, as part of the input, to the hidden layer of the next time step to preserve previous information. After temporal features are extracted, the data are transmitted to fully connected layers to perform the classification or prediction, as with the previously explained models.
  To extract these temporal features, the model maps the input data to a sequence of hidden parameters of the network. This leads to an output series of activation by implementing (3):
  (3) $$h\left(k\right)=\sigma (W_{hx}x\left(k\right)+h\left(k-1\right)W_{hh}+b_{h})$$
  where σ is the used nonlinear activation function for the DNN, h(k) represents the hidden parameters of the network, x(k) represents the input data, $b_{h}$ is the bias vector of the hidden layer and *W* represents the weights of the kernels. These weights can be divided into two sets of weights: input layer $W_{h}x$ and hidden layers $W_{h}h$.
  The input data for this model are a time series that includes temporal features, which in our case are the 3D coordinates values. These values can be studied to extract the evolution of the movement (direction in 3 axes as well as the speed). This DNN structure has been analyzed in the literature for gesture recognition as well as trajectory prediction, due to its capabilities of extracting features from movements [4,6,7].
- Convolutional LSTM. This model, often called ConvLSTM, is a variation of the previous LSTM model that includes convolutional layers. These initial convolutional layers are used to extract non-temporal features in a previous step. To do so, this structure uses convolutions to study the input data executing convolutions at each gate in the LSTM structure rather than using matrix multiplications typical of the dense layer approach in the traditional LSTM structure. Because of this, apart from time series, image series can be studied with this algorithm to extract information about the time evolution of the images. However, non-image data type inputs can also be used in case this feature extraction step is desired as in this work, where 3D coordinate data will be used as input for this model. However, since the ConvLSTM studies the time evolution of the trajectory, to ensure the length of the characters is always the same, a number of 0s were included at the end of some samples to achieve the desired length.
  This DNN structure, as the previously mentioned ones, was selected due to the high-accuracy results achieved in the literature for gesture recognition and trajectory prediction tasks [6,35]. One of the possible reasons for its good performance results is the fact that this model can extract high-level features such as movement direction before studying its time evolution, leading to a more logical feature study pipeline.

The configuration of each of these models for the specific application researched in this work, as well as a deeper description of the gathered data for this task, are presented in the next section.

## 4. Experiment Definition

### 4.1. Dataset

To the best of our knowledge, there is no public dataset available. So far, only Chen H. et al. [13] have created an air-writing system based on ultrasound technology, using their own dataset. That work was based on an active ultrasound array location, instead of the passive approach described in this work, making not possible the use of this database as input in our system for comparison purposes. Consequently, a new ultrasound data dataset was recorded for this experiment.

**Figure 6:**
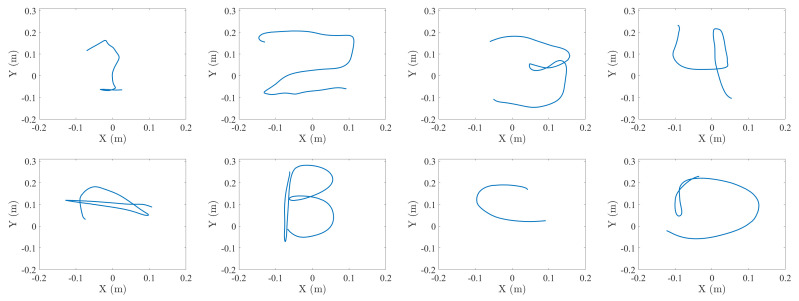
Samples of series of 3D coordinates from the recorded gestures.

This dataset, recorded to test the proposed system, is composed of a series of 3D coordinates where each of these series represents one sample of the studied gestures. These gestures are the digits “1”, “2”, “3” and “4”, as well as the characters “A”, “B”, “C” and “D”, as shown in Figure 6. Due to the characteristics of the studied gestures, the length of the 3D coordinates series varies. The length of each gesture is in the range of 5–8 s except for the gesture “C”, which, due to the simplicity of performing it, takes between 3 and 7 s. The labeling and data separation tasks have been executed manually to ensure the presence of a single gesture in each gesture sample. The gesture end detection was based on detection of the lack of new position information, which would mean the target cannot be located anymore.

**Figure 7:**
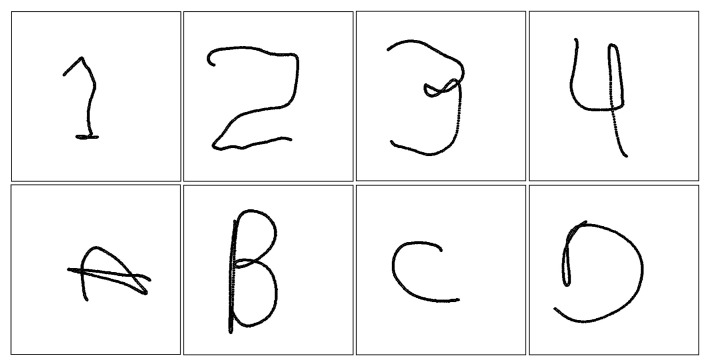
Samples of images generated from the studied gestures.

Using the previously explained time-series dataset, a 2D image dataset was generated. These images, of dimensions 100 × 100 pixels, are the projection of the gestures in the XY plane, as shown in Figure 7.

**Figure 8:**
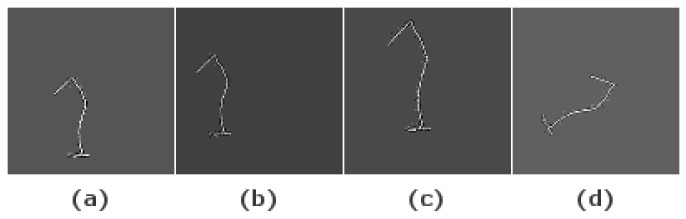
Images of original “1” image (**a**), translation (**b**), translation-scaling (**c**) and rotation (**d**).

Initially, 40 samples were recorded of each possible gesture. During the data recording sessions, the intraclass diversity of the gestures was ensured to generate a dataset that represents a large number of possibilities and scenarios. Then, multiple data augmentation techniques were applied to generate synthetic data in order to have a large enough dataset to train the DL models. An example of the resulting images after applying this technique is shown in Figure 8. The used data augmentation techniques are:

- Gesture translation (Figure 8b): The center positions of the initial gestures were not constant but they were always near the center of the image. To include more positions in the dataset, all the gesture were translated so their centers are located in the center position in the XY plane. After this step, a random translation is performed in the X- and Y-axis or only in one of them.
- Gesture scaling (Figure 8c): The gestures were scaled within a random percentage in the interval 20–50% to generate a more variate dataset. Since the data are represented in two dimensions, each time that scaling was applied, a random variable controls if the scaling was performed in one of the axis or in both as well as the scaling factor for each axis. Consequently, uniform scaled images as well as anisotropic scaled images are included in the dataset.
- Gesture rotations (Figure 8d): The images were rotated at a random angle in the interval 1–359° to generate positions different from the original. As a result of this, all writing directions are included in the generated dataset.

Gesture translation, scaling and rotation were selected as the data augmentation techniques due to the spacial characteristic of the initial data. These techniques generate synthetic data based on the original gestures to extend the initial dataset. For example, as a result of the rotation technique, the dataset includes gestures in all orientations to ensure that the system can recognize gestures no matter their orientation. The same procedure would apply to translation and scaling, which makes the system independent of the center position and size of the gestures, respectively.

The dataset was augmented to 27,670 samples where 5539 (which includes 20% of the original samples as well as the synthetic data generated based on those original gestures using data augmentation techniques) were used for testing purposes and 22,131 for training. Consequently, the data (original and synthetic) were not shared between train and test dataset to correctly measure the accuracy results in the test data. These test and train sub-datasets were equally split between the image and 3D coordinate data to ensure a correct comparison of the accuracy results among the studied algorithms.

### 4.2. Deep Neural Networks Configuration

Each of the researched algorithms for the classification task studied in this paper was tuned and trained to fit the application. Therefore, their final structure and characteristics are further commented on in each of the following subsections.

#### 4.2.1. Convolutional Neural Network

**Figure 9:**
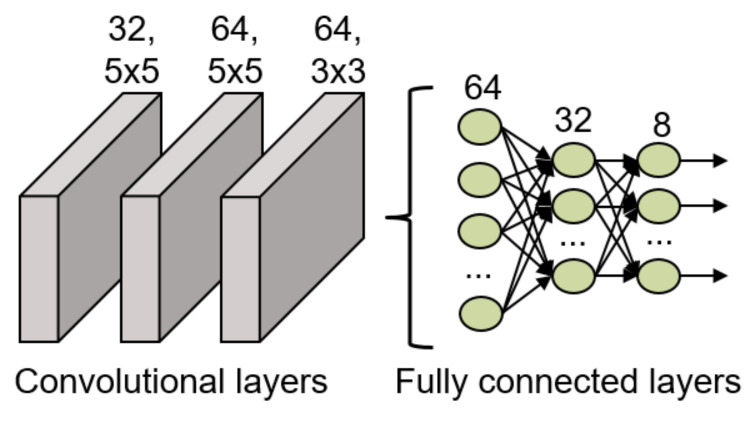
CNN structure implemented for the gesture recognition.

The final structure of this model is shown in Figure 9, where it is possible to observe that it has 3 convolutional layers to extract relevant features from the input data. The first layer has 32 filters of dimensions 5 × 5, the second layer has 64 filters of dimensions 5 × 5 and the final convolutional layer has 64 filters of dimensions 3 × 3. After these convolutional layers, a flatten layer and 3 fully connected layers (64, 32 and 8 neurons, respectively) are included in the network structure to classify the features into the 8 possible gestures. Between all the layers, batch normalization layers have been included to ensure the normalization of the data is not lost during the data study. All the layers included in this network use the ReLU activation function except for the last fully connected layer which uses the softmax activation function for the final classification.

#### 4.2.2. Convolutional Autoencoder

The training of this model takes place in two steps:

- The two main components of the autoencoder are trained to reconstruct as output, the images provided as input. The structure of this model consists of three 2D convolutional layers for the encoder and another three identical and mirrored layers for the decoder. In the encoder, the first layer has 128 filters of dimensions 5 × 5, the second layer has 64 filters of dimensions 3 × 3, and the final convolutional layer has 32 filters of dimensions 3 × 3.
  The internal code layer, which provides the embedding, consists of a max 2D pooling applied on the 32 filters. All these convolutional layers use the ReLU activation function, except for the last layer of the decoder, which employs a sigmoid activation function for the nonlinear image reconstruction. The used loss function is the binary cross-entropy while the optimizer is Adam. The application of the autoencoder enables the reduction of dimensionality from 10,000 (100 × 100) corresponding to an image, to only 1296 (6 × 6 × 36) values, which represent the embedding space dimension.
- After the autoencoder training, the encoder part is extracted and kept frozen for training, so that it can be used as a feature extractor without further parameter tuning. A flatten layer and two dense layers consisting of 32 and 8 neurons respectively are then connected to the model. The parameters of the fully connected layer are trained so as to associate the information extracted from the encoder with the respective labels of the drawn characters. The first dense layer uses the ReLU activation function while the second one uses the softmax activation function for the categorization purpose.

**Figure 10:**
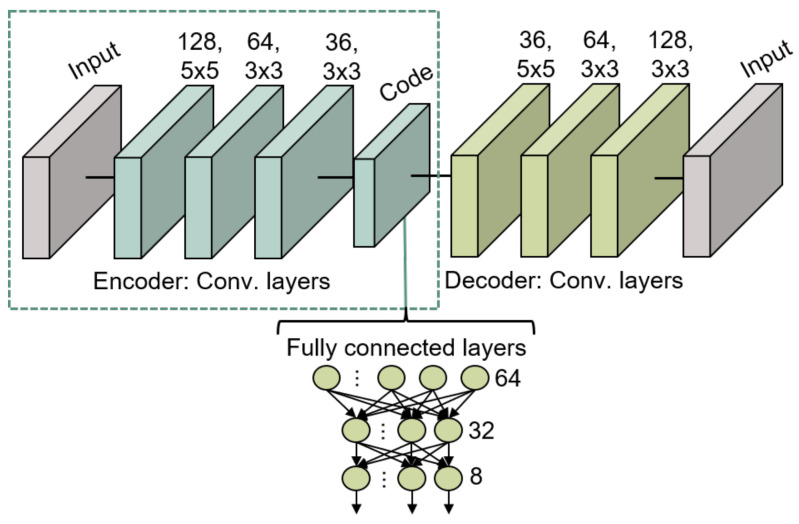
Convolutional Autoencoder structure for gesture recognition.

The model in its ensemble (Convolutional Autoencoder and Fully Connected) is shown in Figure 10.

#### 4.2.3. Long Short-Term Memory Neural Network

**Figure 11:**
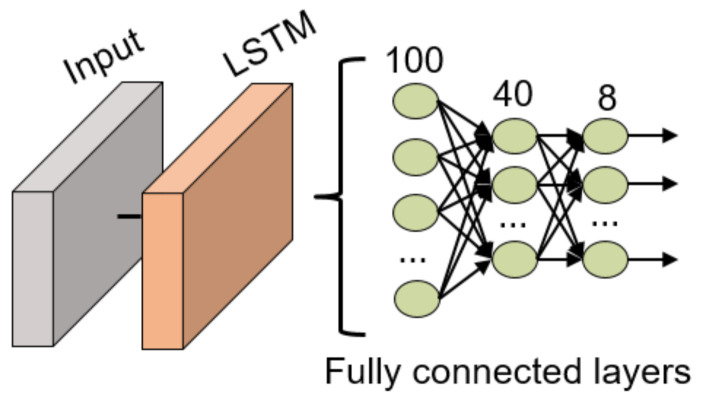
LSTM NN structure for gesture recognition.

The structure of this network is an LSTM layer with 100 units, with a time-step of size 10, followed by 3 fully connected layers (100, 40 and 8 neurons in each layer, respectively) with batch normalization layers between the fully connected layers. All these fully connected layers use the ReLU activation function except for the last layer which uses the traditional softmax activation function for classification tasks. The structure can also be visualized in Figure 11.

#### 4.2.4. 1D Convolutional LSTM Neural Network

**Figure 12:**
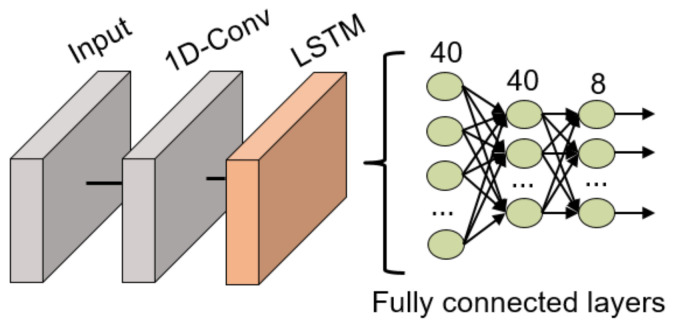
1D-ConvLSTM NN structure for gesture recognition.

The network structure is similar to the previously commented LSTM DNN but it includes a 1D convolutional layer at the beginning of the network to extract relevant features that later can be studied over time, as shown in Figure 12. This convolutional filter has a dimension of 128 × 3. Following the convolutional layer, an LSTM layer with 100 units is in charge of studying the time evolution of the data and 3 fully connected layers (40, 40 and 8 neurons, respectively) to classify the data. All these fully connected layers use the ReLU activation function except for the last layer which uses the traditional softmax activation function for classification tasks. Between all layers, batch normalization layers have been included to ensure the normalization of the data is not lost during the model training.

## 5. Results

The results of the studied classification algorithms when using the test dataset are presented in Table 2. The compared parameters are the accuracy, number of parameters and the latency of the models to provide information regarding their suitability for the tasks as well as the complexity and size of the models.

It is important to remark that all the latency measurements have been executed in the same device (Intel core i5 CPU) in order to be able to compare the latency results. Similarly, test datasets (images and 3D coordinates) contain the same samples.

Table 2 shows how the accuracy achieved by the CNN, ConvAutoencoder and ConvLSTM classification algorithms are quite similar since they all achieve accuracy of above 97.39% while the LSTM algorithm achieves accuracy of 83.25%. The model that achieved the best accuracy results is the ConvLSTM model with 99.51%. However the CNN and ConvAutoencoder achieved similar results with a difference of a 2.02% and 1.23% lower accuracy respectively. The LSTM model achieved the lowest accuracy among the studied algorithms with a different respect the ConvLSTM model of a 16.26% in the accuracy results. This may be the result of the complexity of the dataset when study as individual numerical values in a time series in comparison with an image where the information of the gesture is easier to extract. Regarding the number of parameters and latency, the CNN model has the larger number of parameters (1,730,472 parameters), which is one or two orders of magnitude higher than the rest of the models. The LSTM model only has 56,868 parameters. This shows how there is a trade-off between the size of the model and the accuracy, apart from the network structure. However, even when the CNN model has the larger number of parameters, it still achieves latency results comparable with the rest of the models and even faster than the ConvLSTM and LSTM models. Nevertheless, the fastest model among the studied ones is the ConvAutoencoder that only requires 45.5 milliseconds to generate an output from an input data. The rest of the models require a similar time between 63.43 and 71.90 milliseconds.

**Figure 13:**
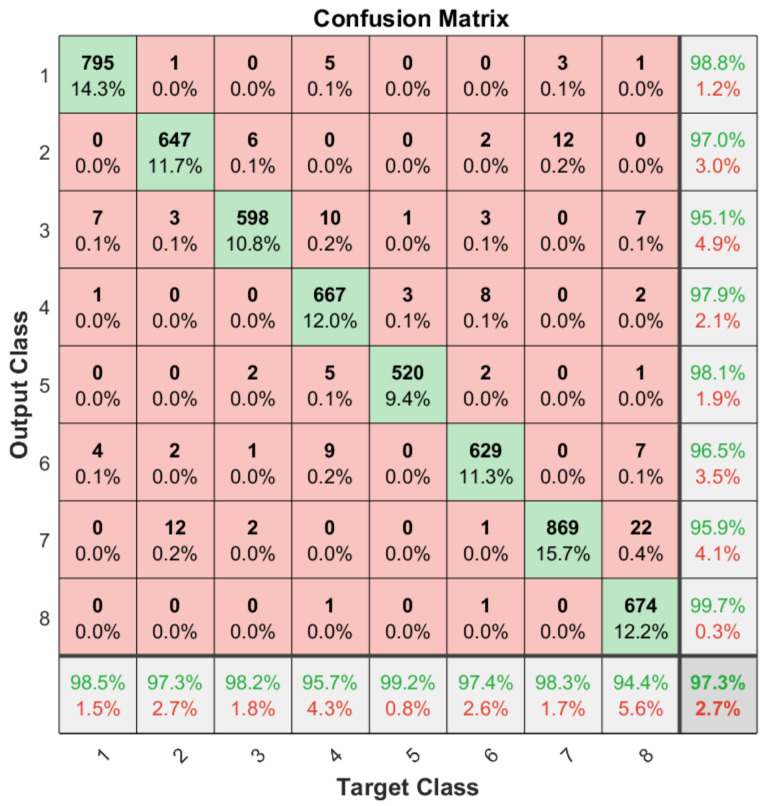
Confusion matrix generated using the CNN algorithm.

**Figure 14:**
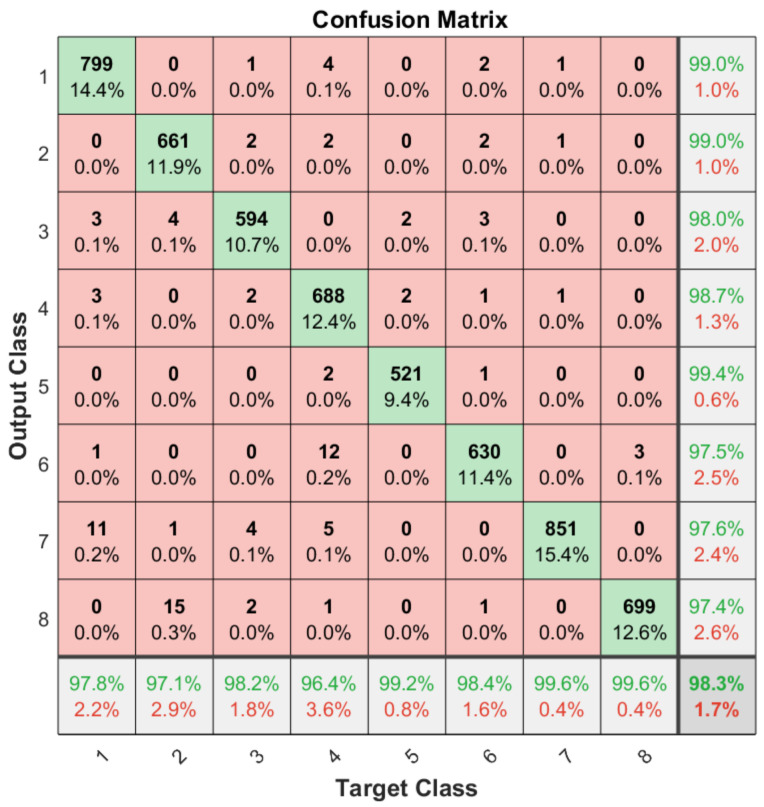
Confusion matrix generated using the ConvAutoencoder algorithm.

**Figure 15:**
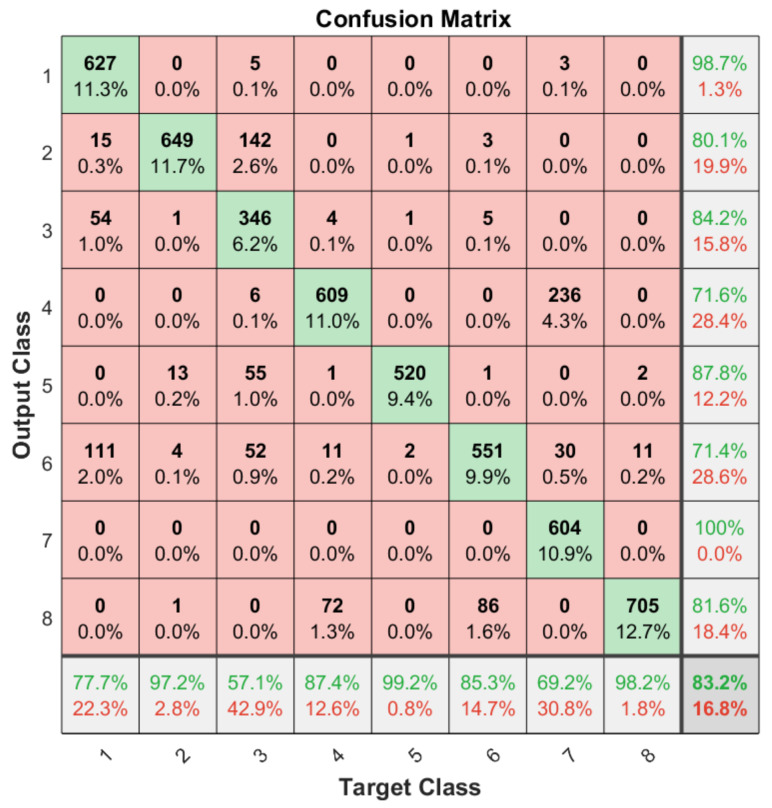
Confusion matrix generated using the LSTM algorithm.

**Figure 16:**
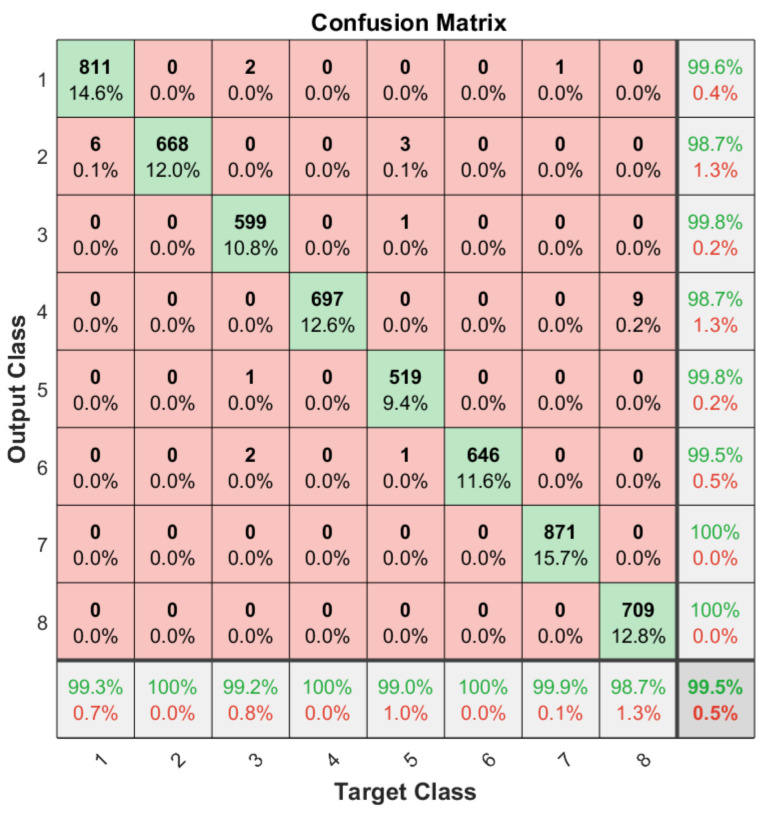
Confusion matrix generated using the ConvLSTM algorithm.

Apart from the general accuracy achieved by the studied models, the accuracy for each of the individual classes is shown in Figure 13, Figure 14, Figure 15 and Figure 16. The labels in these tables have been codified where gestures “1” to “4” are represented with the labels 1 to 4 and the characters “A” to “D” with the labels 5 to 8. These figures show how the accuracy of the classes are well balanced in the studied algorithms except for LSTM, where the gesture “1” and “C” achieved an accuracy under the average. At the same time, in Figure 13 we can see how most of the CNN misclassification errors are located in the gestures “2”, “C” and “D”. This may be because these three gestures have similar curves that can lead the algorithm to a misclassification in some cases. In the rest of the studied algorithms, the error distribution among classes does not indicate a clear misclassification between specific classes since errors are equally distributed among all of them.

It can be observed how the ConvLSTM model has the best performance taking into account the trade-off among its latency, the number of parameters and classification accuracy (99.51%), especially when comparing it with the LSTM model that also studies the temporal evolution of the data. As regards models that require the whole image as input, the ConvAutoencoder provides better results than the CNN in terms of latency, number of parameters and accuracy. This might be due to the efficient compression of relevant features performed by the ConvAutoencoder that leads to a better classification accuracy.

After the comparison of the performance of the studied classification algorithms, a comparison of our results with other technologies and authors is shown in Table 3. This table shows how multiple technologies are being tested for air-writing tasks. Even when different technologies are used, such as radar, ultra-wide-band and ultrasound, the target detection can be calculated based on similar techniques. This is because these technologies calculate the position of the target by measuring the time difference between the transmission of a signal and the reception of its echo. Consequently, the classification algorithms are compared in Table 3 rather than the technique for the data gathering. At the same time, it is important to remark that, since the technologies, gesture number and platform are different, this comparison should be understood as a general observation to gain a deeper understanding of the state of the art rather than a direct comparison among techniques.

Among the compared techniques, the most popular are the DL techniques such as CNN or LSTM due to their high-performance results for classification tasks. Only one of the compared techniques is based on a different approach, the Order-Restricted Matching (ORM) classification algorithm. This algorithm, differently from the rest of the algorithms, is not trained in advance but features are extracted and later compared directly with a feature template for each of the possible characters. The sequence with the minimum accumulated distance between the features and the template feature is selected as the classification result.

However, even if different technologies and approaches are used for this task, similar accuracy results are achieved. All the compared techniques have an accuracy between 96.31% and 99.7%. Among these techniques, the one that provides the highest accuracy results is the Radio CNN (99.7%). Nevertheless, if the latency of the system is taken into account, the ORM Ultrasound technique may provide a best performance since the accuracy difference compared to the Radio CNN is 3.39% but it achieves a latency reduction by a factor of 29 times. Our studied ConvAutoencoder could be considered as a middle point between these two extreme cases since it achieves an accuracy of 98.28%, higher than the ORM technique, and a latency of 45.5 ms, 7.7 ms faster than the Radar DNN.

Another feature that can be compared is the devices integrated into these systems. The techniques based on radar sensors require at least three sensors in order to locate the target in three dimensions. Each of these radar sensors includes a different number of transmitter and receiver antennas, i.e., each of the radars integrated in the system in [6] uses one receiver and one transmitter antenna. In the case of ORM Ultrasound, two arrays of ultrasound sensors are required. The sensors of the first array are used exclusively for transmitting while the ones from the second array are used to receive the echo signals. On the other hand, the system presented in this work only requires one array where one ultrasound transceiver is used to transmit and receive while the other three transceivers are only used for receiving. As a result of this, a smaller number of devices are required in comparison with the rest of the compared techniques while maintaining similar high-performance results.

## 6. Conclusions

An air-writing system, based on one ultrasonic array that includes four transceivers is presented in this work. The system determines the point-to-point distance to the target from the ToF. Those calculated distances are fed to the positioning algorithm to extract the 3D position of the target, and determine the trajectory as a successive series of points equally spaced in time.

To test this system, a dataset containing eight gestures (four letters and four numbers) was recorded. These raw data were then filtered and preprocessed to generate a dataset for gesture classification. Multiple algorithms were researched for this paper to study this dataset. Since the original dataset was a time-series of 3D coordinates, two different approaches were studied to analyze the data: time evolution algorithms (LSTM and ConvLSTM) and image classification algorithms (CNN and ConvAutoencoder).

It is shown in this paper how these algorithms provided high accuracy, whereby the best result was extracted from the ConvLSTM, with 99.51% accuracy and 71.01 milliseconds of latency, when studying time-series of 3D coordinates. Among the algorithms based on images, the ConvAutoencoder provided the best results with a latency of 45.50 milliseconds and an accuracy of 98.28%. Consequently, we can conclude that the proposed system could be implemented in multiple ways so the recognition algorithm can fit the desired platform/scenario.

## Figures and Tables

**Table 1:** Parameter settings used for the transceivers’ actuation and data acquisition.

Parameters Setting	
Actuation pulse frequency (Fc)	30 kHz
Number of pulses	6
Pulse repetition interval	20 ms
Sampling frequency (Fs)	200 kHz

**Table 2:** Comparison of the studied classification algorithms for the gesture classification.

Algorithm	Number of Parameters	Latency (ms)	Accuracy
CNN	1,730,472	63.43	97.39%
ConvAutoencoder	184,396	45.50	98.28%
LSTM	56,868	71.90	83.25%
ConvLSTM	99,960	71.01	99.51%

**Table 3:** Comparison of state-of-the-art techniques for air-writing.

Studies	No. of Characters	Accuracy	Latency (ms)	Method	Hardware
ORM Ultrasound [13]	26	96.31%	1.8	Order-restricted matching (ORM) classifier	2 ultrasound arrays
Radar DNN [6]	15	98.33%	–	ConvLSTM-CTC	3 radars
Radio CNN [8]	10	99.7%	52.2	CNN	3 radars
This work	8	99.51%	71.01	ConvLSTM	1 ultrasound array
This work	8	98.28%	45.5	ConvAutoencoder	1 ultrasound array

## Data Availability

Data sharing is not applicable to this article.
